# Convention of Medical Teachers, Held in Washington, D. C., April 29, 1870

**Published:** 1870-06

**Authors:** 


### CONVENTION OF MEDICAL TEACHERS.

[We are indebted to the *Philadelphia Reporter* for the following account, which the interest felt by various persons in the subject induces us to insert, although it is little more than a point in chronology.—Ed.]

The delegates from the various medical colleges of the United States assembled in convention on Friday, April 29, in the hall, corner of Tenth and E streets, Washington, D. C.

The object of the convention is to consider the improvements that may be suggested for the system of medical education.

Profs. Bemiss, Stillé, and Moore, were appointed a committee on credentials.

The examination of credentials occupied some time, and it was found that the representation was as follows: New Orleans School of Medicine, Samuel Logan; Howard University, D. C., Robert Reyburn and Silas Loomis; University of South Carolina, A. A. Talley, John T. Darby; Detroit Medical College, E. W. Jenks; Missouri Medical College, J. S. Moore; Chicago Medical College, N. S. Davis; Medical Department Georgetown College, J. H. Thompson; Willamette University, Oregon, Horace Carpenter; University of Louisiana, S. M. Bemiss; Jefferson Medical College, Philadelphia, S. D. Gross; University of Pennsylvania, F. G. Smith, Alfred Stillé; St. Louis Medical College, J. B. Johnson; Washington University of Baltimore, Charles W. Chancellor; University of Louisville, David W. Yandell.

Profs. Cox, Smith, Yandell, Logan, and Davis, were then appointed a committee to nominate officers.

The committee, after a short absence, reported the following named gentlemen, who were unanimously chosen to fill the offices for which they were designated by the committee:

*President*—S. D. Gross, of Philadelphia.
*Vice President*—D. W. Yandell, of Kentucky.
*Secretary*—N. S. Davis, of Chicago, Illinois.

The minutes of the last convention held at New Orleans were read, and the report of the committee of that convention made the basis for the action of this.

The convention then adjourned till 10 A. M., April 30th.

Additional credentials were presented as follows: Kansas City Medical College, John M. Forrest, A. P. Larkford; Missouri Medical College, A. Hammer; University of Nashville, W. K. Bowling and W. F. Briggs.

Professor N. S. Davis offered several rules for governing the proceedings, which were adopted.

Dr. Davis offered a resolution that the several propositions adopted by the Convention at Cincinnati, in 1867, be taken up separately in the order in which they stand in the printed report. Adopted.

The first proposition taken up is as follows:

First. That every student applying for matriculation in a medical college shall be required to show, either by satisfactory certificate or by direct examination by a committee of the faculty, that he possesses a knowledge of the common English branches of education, including the first series of mathematics, the elements of the natural sciences, and sufficient knowledge of Latin and Greek to understand the technical terms of the profession, and that the certificate presented (of the result of the examination thus required) be regularly filed as a part of the records of each medical college.

Professor Logan offered an amendatory resolution that recommendations to the different faculties of the medical colleges are not binding until ratified by the several institutions.

After some discussion the resolution was lost.

Professor Moore, of St. Louis, moved to strike out all after the words "common education," and said that in institutions not represented here the recommendations would be impossible, and he thought the tendency in the Western institutions would be to close them up. We exclude men from our colleges simply because they are not classical scholars. This was wrong, as in many instances our best physicians are without a classical education.

Considerable discussion ensued, in which Professors Yandell, of Kentucky; Hammer, Davis, Moore, Cox, Loomis, Stillé, and Reyburn, took part.

Professor Hammer moved an amendment to the amendment offered by Dr. Moore, to the effect that the words relating to the Latin and Greek languages be stricken out, and all else retained.

Professor Yandell offered resolutions as a substitute, which, after a protracted debate, were rejected. The amendment to the amendment was withdrawn, and the question recurred on the original resolution, which was debated at length, when a vote was taken and adopted, reconsidering the vote by which Professor Stillé's substitute was lost.

A motion was made to adopt the substitute, when debate again occurred.

Professor Davis advocated the adoption of the substitute, saying that he promised, if the schools of Boston, New York, and Philadelphia, would adopt the recommendations, and put them into operation, that the whole West would follow suit at once. (Great applause.)

The substitute of Professor Stillé was then adopted as a substitute for all the propositions before the convention. It is to the effect, "that the propositions adopted in 1867 by the convention of delegates from medical colleges, embodying a system of collegiate medical education, are in the highest degree commendable, and if they could be generally carried into effect would tend to elevate the medical profession. That the requirements for the degree of Doctor of Medicine must be practically determined by each medical college for itself, by the average attainments of its students, and by other considerations of which it alone can judge, and that, consequently, while abstaining from all attempts at dictation, this convention reiterates in the strongest manner its desire that the several medical colleges will, in the changes from time to time made by them in the curriculum of study, endeavor to conform them to the general plan which was recommended by the convention of 1867, and adopted in the same year by the American Medical Association."

The National Convention of Medical Colleges passed the preamble and resolutions offered by Professor Logan, setting forth that as this convention has failed to secure the assent of the majority of the regular medical colleges of the United States to the system of improvement in medical education recommended at its last session, and as it is the opinion of this convention that the best means by which a judicious system of gradual improvement in medical education can be inaugurated by the medical colleges of this country will be found in the associated action of such colleges as will unite for that purpose,

*Resolved*, First. That a committee of nine be appointed, whose duty it shall be to communicate with the faculties of the medical colleges in the United States, with the view to ascertain how many and which may be willing to become members of an association of medical colleges, having for its prime object the improvement of medical education.

Second. That the chairman of said committee be instructed, as soon as he shall have received affirmative replies from the regular colleges, to inform each faculty so consenting of the fact, and to request that each faculty elect one or more delegates to the convention on the Friday before the day appointed for the meeting of the American Medical Association in 1871, and at the place of meeting chosen by that body, said delegates to be fully authorized to pledge their respective faculties to whatever definite plans of improvement in medical education may be adopted by the body in convention.

Third. It is hereby recommended that said delegates organize themselves, in behalf of their respective institutions, into a permanent association of medical colleges for the above mentioned object, and with the view of co-operating with the American Medical Association and the profession at large to accomplish so desirable an end.

Fourth. That Professor N. S Davis, the chairman of the committee appointed by this body at its last session to communicate with the medical colleges on the same subject, be made chairman of this committee, and that the committee be authorized to fill any vacancies which may occur in its ranks.

The chair appointed the following as the Committee: Professor N. S. Davis, of Illinois; Samuel Logan, of New Orleans; A. Hammer, of St. Louis; T. Parvin, of Louisville, S. D. Gross, of Philadelphia; G. C. Blackman, of Cincinnati; G. G. Shattuck, of Boston, and A. C. Post, of New York.

The Convention adjourned *sine die.*

